# Dr. Robert James Carter

**Published:** 1909-05-01

**Authors:** 


### OBITUARY.

The death is announced of Dr. Robert James Carter, M.D. Lond., M.R.C.S., D.P.H., at St. John's Wood, at the age of 44. Dr. Carter was a physician at the Western Skin Hospital and had been a house-surgeon at the Soho Male Lock Hospital, resident medical officer at the Royal Hospital for Women and Children, clinical assistant at the Evelina Hospital, house surgeon at King's College Hospital, and surgeon to the Clan Line Steamship Company.

